# HIV Status Disclosure and Retention in Care in HIV-Infected Adolescents on Antiretroviral Therapy (ART) in West Africa

**DOI:** 10.1371/journal.pone.0033690

**Published:** 2012-03-21

**Authors:** Elise Arrivé, Fatoumata Dicko, Hind Amghar, Addi Edmond Aka, Hélène Dior, Belinda Bouah, Mariam Traoré, Patricia Ogbo, Hortense Aka Dago-Akribi, Tanoh Kassi F. Eboua, Kouadio Kouakou, Haby Signate Sy, Ahmadou Alioum, François Dabis, Didier Koumavi Ekouévi, Valériane Leroy, for the Pediatric IeDEA West Africa Working Group

**Affiliations:** 1 INSERM, ISPED, Centre INSERM U-897-Epidemiologie-Biostatistique, Bordeaux, France; 2 Université Bordeaux, ISPED, Centre INSERM U-897-Epidemiologie-Biostatistique, Bordeaux, France; 3 Hopital Gabriel Touré, Bamako, Mali; 4 Centre de Prise en charge de Recherche et de Formation, Abidjan, Côte d'Ivoire; 5 Hopital Albert Royer, Dakar, Sénégal; 6 Centre Hospitalier Universitaire Yopougon, Abidjan, Côte d'Ivoire; 7 Centre Intégré de Recherches Biocliniques, Abidjan, Côte d'Ivoire; 8 PACCI, Abidjan, Côte d'Ivoire; Vanderbilt University, United States of America

## Abstract

**Objective:**

We assessed the effect of HIV status disclosure on retention in care from initiation of antiretroviral therapy (ART) among HIV-infected children aged 10 years or more in Cote d'Ivoire, Mali and Sénégal.

**Methods:**

Multi-centre cohort study within five paediatric clinics participating in the IeDEA West Africa collaboration. HIV-infected patients were included in this study if they met the following inclusion criteria: aged 10–21 years while on ART; having initiated ART≥200 days before the closure date of the clinic database; followed ≥15 days from ART initiation in clinics with ≥10 adolescents enrolled. Routine follow-up data were merged with those collected through a standardized *ad hoc* questionnaire on awareness of HIV status. Probability of retention (no death or loss-to-follow-up) was estimated with Kaplan-Meier method. Cox proportional hazard model with date of ART initiation as origin and a delayed entry at date of 10th birthday was used to identify factors associated with death or loss-to-follow-up.

**Results:**

650 adolescents were available for this analysis. Characteristics at ART initiation were: median age of 10.4 years; median CD4 count of 224 cells/mm^3^ (47% with severe immunosuppression), 48% CDC stage C/WHO stage 3/4. The median follow-up on ART after the age of 10 was 23.3 months; 187 adolescents (28.8%) knew their HIV status. The overall probability of retention at 36 months after ART initiation was 74.6% (95% confidence interval [CI]: 70.5–79.0) and was higher for those disclosed compared to those not: adjusted hazard ratio for the risk of being death or loss-to-follow-up = 0.23 (95% CI: 0.13–0.39).

**Conclusion:**

About 2/3 of HIV-infected adolescents on ART were not aware of their HIV status in these ART clinics in West Africa but disclosed HIV status improved retention in care. The disclosure process should be thus systematically encouraged and organized in adolescent populations.

## Introduction

In West Africa, children infected with HIV through mother-to-child transmission, once diagnosed and in care, live longer and reach adolescence, because of greater access to antiretroviral therapy (ART) drugs observed in West Africa in the past decade [1], [2]. Adolescence is characterized by remarkable physical, mental and social changes and difficulties. In addition, adolescents, starting sexual activity are at high risk of HIV acquisition and transmission in areas of generalized epidemic [2].

Emotional and behavioural disorders have been described in Africa throughout adolescence. Musisi et al reported that, among 82 Ugandan HIV-infected adolescents, 98% were orphans, 51% reported psychological distress and 17% attempted suicide [3]. In a study in Zambia, about 40% of 127 HIV-infected adolescents reported mental health problems [4].

Virological failure has also been observed among adolescents [5], 6,7 such as in a US series where it was observed in 67% of 154 adolescents, mainly associated with the lack of adherence and suboptimal ART use [8]. Treatment adherence may be one of the critical issues for therapeutic long-term success in adolescents. Problems of adherence were indeed noticed, not only in an American study, in 40% out of 396 adolescents [9], but also in Southern Africa in more than 60% of the adolescents surveyed [5].

Disclosed HIV status has been identified as one of the factors associated with better adherence [10], [11]. However, the prevalence of disclosure in children and adolescents varies by setting and according to the age of the patients, from 13 to 60% in children between 5 to 17 years from Asia or Southern-Eastern Africa [4], . While also essential for the secondary prevention of HIV transmission, disclosure for adolescents may accentuate emotional and behavioural disorders, familial conflicts or social stigma perception and may thus jeopardize confidentiality [20], [21]. HIV status disclosure of youths represents a challenge for the family and for medical staff [22], [23], [24].

The disclosure process in adolescents is poorly documented outside Southern Africa and it is not known whether it may influence their clinical response to ART. The main objectives of this study were thus (1) to estimate the frequency of HIV disclosure in an adolescent population in West African settings; (2) to assess the effect of disclosed HIV status to HIV-infected adolescents on ART clinical outcomes (death and loss-to-follow-up).

## Methods

### Ethics statement

The IeDEA West Africa collaboration project received ethical approval from the Bordeaux University Hospital Institutional Review Board. A waiver for informed consent was given as this data was collected from routine patients' charts.

### Study design

We conducted a retrospective study nested in a multi-centre prospective cohort network of paediatric clinics participating in the International epidemiological database to Evaluate AIDS (IeDEA) West Africa collaboration (http://mereva.net/iedea). This collaboration was initiated in 2006 and currently involves 11 pediatric HIV/AIDS clinics spread over seven countries: Benin, Burkina Faso, Côte d'Ivoire, Ghana, Mali, Senegal and Togo [25]. However, as the centers from Côte d'Ivoire, Mali and Senegal followed-up about 90% of all adolescents from this collaboration, the present study was therefore restricted to these countries, including three centers in Abidjan, Côte d'Ivoire (the pediatric ward of the Teaching Hospital of Yopougon, the Centre Intégré de Recherches Biocliniques (CIRBA), the Centre de Prise en charge de Recherche et de Formation (CePReF); the pediatric ward of Gabriel Toure Hospital in Bamako, Mali and the pediatric ward of Albert Royer Hospital in Dakar, Sénégal.

### Study sample

In each pediatric clinic participating in the IeDEA West Africa collaboration, clinical forms were available for recording HIV activities and an electronic database for data entry. All HIV-infected children (positive viral load test at <18 months or positive serology at ≥18 months) aged <21 years with documented gender and antiretroviral drug regimen were included. These HIV-infected children were typically seen in clinics at least every three months, and their CD4 counts were measured twice a year to monitor their immunological response to ART. Routine viral load monitoring was not available in most sites.

The inclusion criteria in the present study were:

Aged 10–21 years during follow-up while on ART
Followed-up in a paediatric clinic with ≥10 adolescents in care
Having initiated ART ≥200 days before the closure date of the local database
Followed-up≥15 days from ART initiation.

Adolescence is usually defined by the World Health Organization (WHO) [26] as the period between childhood and adult age, from 10 to 19 years. In our study we extended the age upper limit to 21 years as this is the age of legal majority in Côte d'Ivoire (18 in the other countries).

Strategies of HIV disclosure varied according to sites: in Dakar, focus group and patient education were proposed. In Bamako, a monthly follow-up for the adolescent and his/her parents was conducted by a dedicated physician. The three sites from Abidjan had a team of psychologists providing psychological support to the children and the caregivers, not only for disclosure, but also for adherence for instance. However, none of the sites had developed local guidelines.

### Data collection

In the context of the IeDEA West Africa collaboration, baseline and follow-up data were recorded in the local databases computerized in the participating clinics and merged together in a larger database (Microsoft Office Access 2003). All data were anonymized using a unique, confidential identification number.

The following data from patients meeting inclusion criteria were extracted from the pediatric central database:

Demographic data: date of birth, gender
ART regimen: type and date of initiation
Biological and clinical data at treatment initiation (or 200 days before): CD4 cell count and CD4 percentage, haemoglobin, CDC and WHO clinical stage
Outcomes: Transfer out, death, CD4 CD4 cell count and CD4 percentage and dates of events

A standardized form was created to extract study specific data from the medical, psychological and social charts available in the patient's files. This form was prepared based on literature review and discussions between the investigators and the staff in charge of disclosure in each clinic. The questionnaire was tested in Abidjan to reach a final version before full implementation in May 2010. It included the following information:

Own HIV status disclosed to the adolescent (“Is the adolescent informed about his/her HIV status?”), and if so,
  - date of disclosure
  - Persons involved: physician, social worker, psychologist…
  - Conditions of disclosure: planned or not, timing (before or after ART initiation)
  - Immediate reaction to disclosure
Individual situation at disclosure or at closure date: living, schooling

The questionnaire was completed by physicians, psychologists or social workers involved in each clinic. Data specific to the study were computerized on each site using Epi-Info software version 5.3.2 and then were merged in the IeDEA West Africa central office in Bordeaux.

### Statistical analysis

To compare the characteristics of adolescents included or not in the analyses, as well as of those with disclosed HIV status or not, Fisher and Chi-square tests and Kruskal-Wallis tests were used for qualitative variables and quantitative variables, respectively.

Probability of death, loss-to-follow-up, and retention in HIV care (patients neither dead nor lost-to-follow-up) was estimated with the Kaplan-Meier method overall and according to the HIV disclosure status. Loss-to-follow-up was defined as the last contact before 21 years of age recorded ≥200 days before the closure date of local database, with no mention of transfer out or death.

The end point was defined as the date of death, loss-to-follow-up, 21^st^ birthday or last contact before the closing date of the database (04/01/2008 for the Teaching Hospital of Yopougon, 05/11/2008 for the CIRBA, 05/11/2008 for theCePReF , 20/12/2008 for the Bamako Hospital, 19/06/2009 for the Dakar Hospital.

To assess factors associated with these risk functions, Cox model was used, with date of ART initiation as origin, censoring at the closure dates of the local databases and with delayed entry at date of 10th birthday for those who initiated ART before 10. All analyses used the center as a cluster variable, taking into account the correlation of the observations within a same center. Missing data (MD) were treated as specific categories except for the variable of interest (disclosed HIV status). Patients with missing data on this variable were excluded.

All analyses were performed with SAS software 8.2 (USA). All p-values were two-tailed. A p-value<0.05 was considered as statistically significant. Variables with a p<0.20 in univariate models were examined in multivariate models and selected thereafter using a backward stepwise procedure. Hazard ratios (HR) and their 95% confidence intervals (CIs) were produced.

## Results

### Sample description

**Figure 1:**
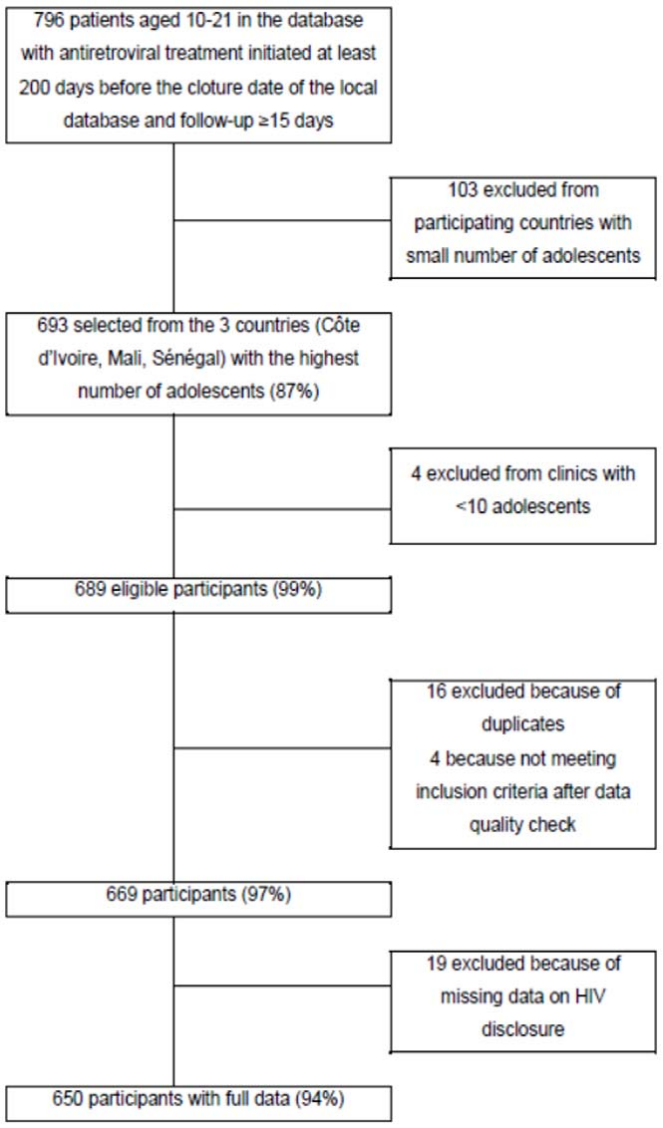
Cohort profile for the adolescent HIV disclosure study. Pediatric IeDEA West Africa (WADA) Collaboration.

The inclusion criteria were met by 669 adolescents (Figure 1) from the five centers selected. Nineteen had missing data on HIV disclosure status and were described separately. Thus, 650 adolescents remained in the analysis, representing 93.8% of the selected population (Figure 1). They had a median age at ART initiation of 10.4 years, a median CD4 count of 224 cells/mm^3^ (46.9% with severe immunosuppression; i.e. CD4<200 cells/mm^3^; 68 with MD), a median haemoglobin of 10.0 g/dL (4.8% with severe anaemia; i.e. blood haemoglobin<6.9 g/dL; 93 MD) and 277 (48.1%) were stage 3 or 4/C at ART initiation (73 MD) (Table 1). Most of them (n = 447; 68.8%) started with a non nucleosidic (NNRTI)-based ART regimen.

**Table 1:** Characteristics of adolescents at ART initiation.

		Abidjan (n = 403)		Bamako (n = 174)		Dakar (n = 73)		Overall (n = 650)	
Variable	Unit	N	Med (IQR)	N	Med (IQR)	N	Med (IQR)	N	Med (IQR)
Age	Years	402	10.7 (8.8–12.8)	174	10.0 (8.4–11.7)	73	9.9 (8.7–13.0)	650	10.4 (8.8–12.7)
CD4	cells/mm^3^	363	283 (78–529)	173	171 (57–325)	46	121 (19–350)	582	224 (63–424)
Haemoglobin	g/dL	356	10.2 (9.0–11.1)	161	9.7 (8.7–10.7)	40	9.0 (7.7–11.0)	557	10 (8.7–11)
Weight	kg	357	24 (19–29)	155	20 (17–25)	69	21 (18–27)	581	23 (19–27)
Stage 3&4/C	N n (%)	350	107 (30.6)	155	106 (68.4)	72	64 (88.9)	577	277 (48.0)
NNRTI	N n (%)	403	271 (67.2)	174	118 (67.8)	73	58 (79.5)	650	447 (68.8)

Pediatric IeDEA West Africa (WADA) Collaboration.

ART: Antiretroviral treatment; NNRTI: non nucleoside reverse transcriptase inhibitor;

N: number; Med: median; IQR: Inter Quartile Range; Min: minimum; Max: maximum.

About 55% of the adolescents (n = 360) started ART after the age of ten. Over a median 35.8 months follow-up period from ART initiation (23.3 months in median from the age of ten), 40 (6.1%) died and 85 (13.1%) were lost-to-follow-up (Table 2). The median age at the endpoint was 13.1 years.

**Table 2:** Follow-up characteristics of adolescents on ART.

		Abidjan (n = 403)		Bamako (n = 174)		Dakar (n = 73)		Overall (n = 650)	
Variable	Unit	Med (IQR)		Med (IQR)		Med (IQR)		Med (IQR) (min-max)	
Follow-up from ART initiation	Months	36.5 (23.6–38.6)		29.1 (16.6–52.1)		45.8 (24.3–67.0)		35.8 (21.2–39.8)	
Follow-up from the age of 10	Months	24.3 (12.5–36.5)		19.0 (9.8–31.6)		25.4 (14.2–49.0)		23.3 (11.3–36.6)	
Age at endpoint	Years	13.3 (11.5–15.3)		12.5 (11.2–14.5)		13.8 (11.7–16.5)		13.1 (11.5–15.3)	
Death	n (%)	19	(4.7)	10	(5.7)	11	(15.0)	40	(6.1)
Mortality incidence from the age of 10	/1000 pers-month	1.97		2.51		4.49		2.49	
Loss-to-follow-up	n (%)	45	(11.2)	35	(20.1)	5	(6.8)	85	(13.1)

Pediatric IeDEA West Africa (WADA) Collaboration.

ART: Antiretroviral therapy.

N: number; Med: median; IQR: Inter Quartile Range; Min: minimum; Max: maximum.

Overall, 187 (28.8%) adolescents knew their HIV status (Table 3). Compared to those not disclosed, adolescents with disclosed HIV status were, at ART initiation:

Older (median age: 11.9 vs 9.8 years, p<0.0001)
More often on NNRTI-based ART (76.5% vs. 65.6%, p = 0.007)
Less often living with their parents (45.9% vs. 57.0%, p = 0.011)
At similar rate of severe immunodeficiency, clinical stage at ART initiation, being orphaned of at least one parent and attending school at the endpoint (p>0.1).

**Table 3:** Characteristics of adolescents at HIV disclosure or last contact.

		Abidjan (n = 403)		Bamako (n = 174)		Dakar (n = 73)		Overall (n = 650)	
Variable		n	(%)	n	(%)	n	(%)	n	(%)
Disclosure	Yes	127	(31.5)	44	(25.3)	16	(21.9)	187	(28.8)
Known date of disclosure	Yes	10/127	(7.9)	40/44	(90.9)	9/16	(56.2)	59/187	(31.5)
Orphanhood	No	98	(24.3)	38	(21.8)	14	(19.2)	150	(23.1)
	One parent	187	(46.4)	61	(35.1)	35	(47.9)	283	(43.5)
	Two parents	100	(24.8)	67	(38.5)	21	(28.8)	188	(28.9)
	Unknown	18	(4.3)	8	(4.6)	3	(4.1)	29	(4.5)
Living with parents	No	162	(40.2)	84	(48.3)	36	(49.3)	282	(43.4)
	Yes	208	(51.6)	86	(49.4)	36	(59.3)	330	(50.8)
	Unknown	33	(8.2)	4	(2.3)	1	(1.4)	38	(5.8)
Schooling	No	54	(13.4)	31	(17.8)	26	(35.6)	111	(17.1)
	Yes	319	(79.2)	108	(62.1)	43	(58.9)	470	(72.3)
	Unknown	30	(7.4)	35	(20.1)	4	(5.5)	69	(10.6)

Pediatric IeDEA West Africa (WADA) Collaboration.

N: number.

The year of disclosure was known for only 59 adolescents (31.5%) (Table 3). This variable was poorly reported in the charts in Abidjan. When known, the median age at disclosure was 15 years (IQR: 14–15; min = 11; max = 19). At the time of disclosure or end point, 471 adolescents (72.5%) were known to be orphaned of at least one parent (biological mother or father) and 330 (50.8%) were known to live at that time with a parent.

### Conditions of the disclosure of the HIV status to the adolescents

Most of HIV disclosures happened after ART initiation and were planned (Table 4). The persons involved in this process varied across the countries. It was mainly conducted by the family, particularly the mother and the father in Abidjan after an interview between the latter and the psychologists, while it was conducted by the health care staff in Bamako and Dakar.

**Table 4:** Characteristics of the process of HIV disclosure of adolescents.

		Abidjan (n = 127)		Bamako (n = 44)		Dakar (n = 16)		Overall (n = 187)	
Variable		n	(%)	n	(%)	n	(%)	n	(%)
Mother involved in disclosure		35	(27.6)	0	(0)	0	(0)	35	(18.7)
Father involved in disclosure		26	(20.5)	1	(2.3)	0	(0)	27	(14.4)
Physician involved in disclosure		4	(3.1)	40	(90.9)	8	(50.0)	52	(27.8)
Psychologist involved in disclosure		6	(4.7)	0	(0)	3	(18.7)	9	(4.8)
Social worker/counsellor involved in disclosure		1	(0.8)	0	(0)	5	(31.2)	6	(3.2
	Other*	62	(48.8)	5	(11.4)	2	(12.5)	69	(36.9)
Conditions of the disclosure	Planned	67	(52.8)	40	(90.9)	9	(56.2)	116	(62.0)
	Not planned	55	(43.1)	0	(0)	5	(31.2)	60	(32.1)
	Not known	5	(3.9)	4	(9.1)	2	(12.5)	11	(5.9)
Timing of disclosure	Before ART initiation	10	(7.9)	0	(0)	2	(12.5)	12	(6.4)
	After ART initiation	107	(84.2)	40	(90.9)	13	(81.2)	160	(85.6)
	Not known	10	(7.9)	4	(9.1)	1	(6.2)	15	(8.0)

Pediatric IeDEA West Africa (WADA) Collaboration.

ART: Antiretroviral therapy.

* Consisted in other persons of the family (uncle, aunt, grand-mother, grand-father, adoptive father or mother), persons from foster care shelters or NGOs (n = 14), nobody involved because of reading the notice (n = 4).

### Effect of the adolescents' knowledge of their HIV status on retention in care

**Figure 2:**
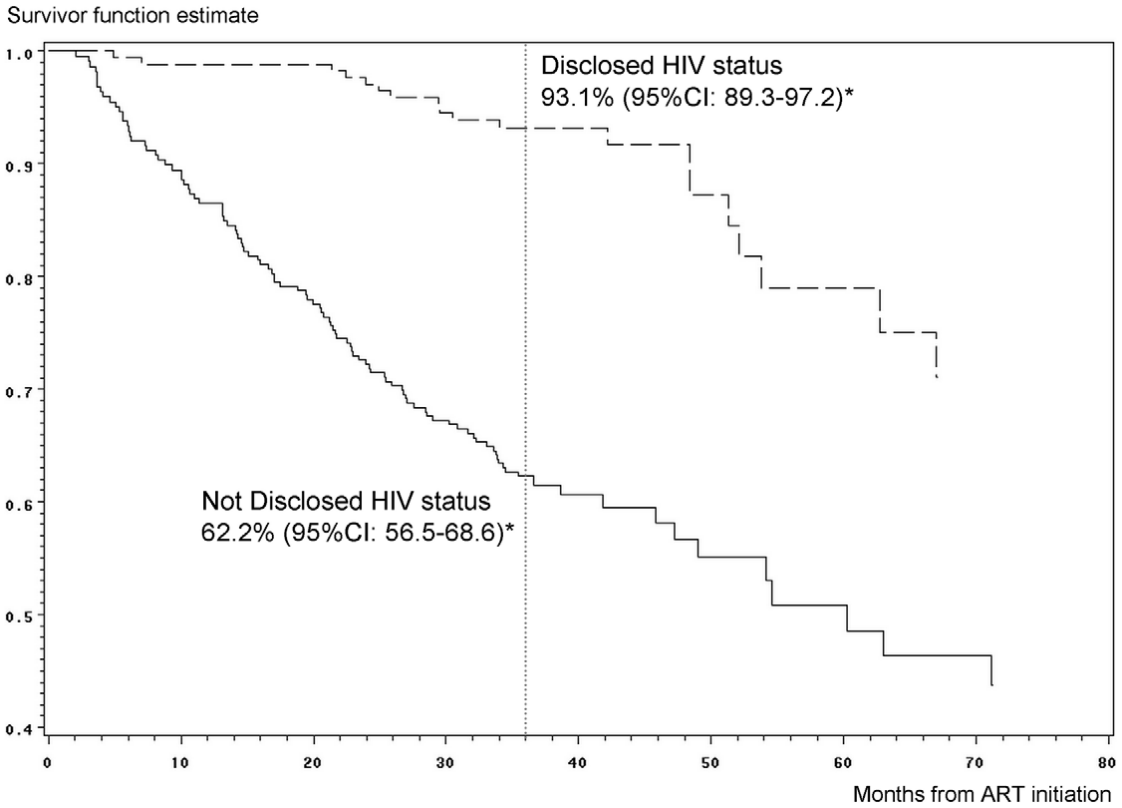
Probability of retention in HIV care while on antiretroviral therapy (ART) according to disclosed HIV status taking into account the delayed entry at age of 10 years (n = 650). Pediatric IeDEA West Africa Collaboration. * Estimation at 36 months from ART initiation.

The overall 36-month probability of retention after ART initation in this sample aged ≥10 was 74.6% (95% CI: 70.5–79.0). It was significantly lower for those whose HIV status was not disclosed (Figure 2). Overall, the 3-year probability of survival was 92.1% (95%CI: 89.4–95.0) and the probability of not being lost-to-follow-up was 80.4% (95%CI: 76.5–84.5). Disclosed HIV status was associated with higher retention in care in multivariate analysis: adjusted hazard ratio (aHR) = 0.23 (95% CI: 0.13–0.39; p<0.0001) (Table 5).

**Table 5:** Crude and adjusted hazard ratios (HR) with 95% confidence intervals (CI) of risk of death or loss-to-follow-up of adolescents after ART initiation (n = 650).

		Crude			Adjusted		
Variables		HR	95%CI	p-value	aHR	95%CI	p-value
Disclosure (ref: no)	Yes	0.23	0.13–0.39	<0.0001	0.23	0.13–0.39	<0.0001
Gender	Male	1.08	0.76–1.55	0.655			
CD4<200 cells/mm^3^ (ref: no)	Yes	1.73	1.17–2.55	0.014	1.65	1.11–2.44	0.017
	MD	1.75	0.99–3.08		1.97	1.07–3.62	
Severe anaemia (ref: no)	Yes	0.74	0.27–2.01	0.363			
	MD	1.34	0.84–2.01				
NNRTI- based regimen (ref: no)	Yes	0.87	0.84–1.13	0.461			
Stage 3&4/C (ref: no)	Yes	1.29	0.88–1.88	0.405			
	MD	1.07	0.57–2.02				
Country (ref: Côte d'Ivoire)	Mali	1.78	1.19–2.66	0.014	1.74	1.15–2.64	0.016
	Sénégal	1.09	0.62–1.93		0.87	0.48–1.57	

Pediatric IeDEA West Africa Collaboration.

All analyses used the center as a cluster variable, taking into account the correlation of the observations within a same center.

HR: Hazard ratio.

aHR: adjusted hazard ratio.

MD: missing data.

ART: Antiretroviral therapy.

NNRTI: non nucleoside reverse transcriptase inhibitor.

Severe anemia: haemoglobin≤6.9 g/dL.

Severe immunosuppression : CD4<200 cells/mm^3^.

### Description of excluded patients with MD on disclosure and sensitivity analysis

There were 19 adolescents excluded because of MD on disclosure status (17 from Abidjan). Three were dead and five lost to-follow-up by 31 months in median after ART initiation after the age of ten.

If all patients with missing data were classified as not-disclosed HIV status, the probability of retention for these patients decreased to 60.8% (95%CI 55.2–67.1), increasing the difference acording to the HIV disclosure status. If all patients with missing data were classified as disclosed HIV positive status, the probability of retention for these patients increased to 89.7% (95%CI 84.4–93.9), decreasing the difference between the two groups but it remained statistically significant, in univariate and multivariate analyses (aHR = 0.28; 95%CI = 0.18–0.44).

## Discussion

In this multicentered cohort study conducted in West Africa, we described a large sample of HIV-infected adolescents who started ART at a median age of 10.4 years. Despite this information was not documented with reliability in this cohort, it is estimated that more than 90% of the children/adolescents acquired HIV through mother-to-child transmission and can be considered as slow progressors [27]. We observed that about two thirds of HIV-infected adolescents on ART beyond the age of ten were not aware of their own HIV status. When the age at disclosure was known, it was delayed around a median of 15 years. We demonstrated a beneficial effect of HIV disclosure on retention in care among adolescents on ART.

Although low, the frequency of disclosed HIV status in our study was higher than previously reported figures in studies conducted in Nigeria, Ethiopia, Thailand and India where the children were younger (mean age = 8–9 years) [13], [17], [18], [28] or Ghana, with a median age of ten [12]. It was similar to other studies conducted in Uganda and Zambia in children aged 12 in median [4], [11] or Thailand (mean age 9.2 years) [15] but a bit lower than an Ivorian study involving adolescents older than 13 years [19]. Indeed, disclosure was often associated with an age>10 or even older [13], [17], [18], [29], [30], supporting our observations. In a qualitative study conducted in South Africa, the age of ten has been reported by health workers as the most appropriate to start having discussion regarding HIV infection [31].

In a US perinatally HIV-infected cohort, HIV disclosure was observed to occur at younger age over time, which may suggest a decline in the perception of HIV stigma [29]. In this industrialized country, the social and medical network may have been strengthened, leading to this condition. In resource-limited settings, despite local interventions, such as peer support groups, or community organizations for social support, stigma and fear of negative reactions or psychosocial outcomes may remain strong barriers to an earlier HIV disclosure to children [4], [12], [15], [17], [22], [32], [33], [34], [35].

Conditions of disclosure varied according to settings. In Abidjan, relatives were encouraged to conduct the disclosure while the medico-social staff was more involved in the other countries. In Nigeria, most of the disclosure was conducted by family, preferably the mother, at home [18]. In Ghana, it was also mostly conducted by caregivers [12], but among those who had not disclosed, one-third wanted to defer to the health workers. Similarly, in Ethiopia, 60% of 193 caregivers interviewed thought that the doctor was responsible for disclosure [17] and in Thailand, 50% of the interviewed caregivers reported the need of assistance from health workers [15]. These findings were supported by a study on health workers' perceptions conducted in a South African setting [31]. In this present study, the sites implemented different strategies to conduct the disclosure process (described in the Methods), which could explain, in part, the differences observed between across the cities in terms of retention. Also, the investigators of the participating centers were aware of the recommendations from the French-speaking association called Grandir [36], [37]. However, they faced difficulties not addressed by these recommendations. For instance, they had problems to manage the caregivers' reluctance to disclose or to make understand to the adolescent what HIV is and its impact on life. They required sharing field experiences to harmonize process and guide local policy on HIV disclosure to be conducted in optimal conditions involving both adolescents and their caregivers. Recent guidelines proposed by the World Health Organization underlined the absence of evidence as to who best can disclose to the child his/her HIV status, caregiver or health care worker with or without specific training [38].

In this large cohort with three-year follow-up, we observed a relatively low death rate but a quite important loss-to-follow up rate, similar to a previous report on clinical outcomes in West African children [25]. In a study conducted in Uganda, including 575 patients starting ART during adolescence, the cumulative survival at 36 months was 90% (95% CI 87.9–93.1) [39]. This figure was similar to the survival rate found in our study where 55% has initiated ART after the age of ten. We found that HIV disclosure was associated with better retention in care as already reported in a Romanian setting among adolescents with a mean age of 13 years [40].

Several limitations could be discussed in our study. First, the main limit of this study is its partially retrospective design and the fact that it relied heavily on the patients' charts, leading to missing data and possible information bias. In particular, the HIV disclosure status was not systematically reported in the patients' charts. However, the forms were filled in by clinic staff, who knew the patients and their family environment quite well and could remember and cross-check who was informed of his/her HIV status and who was not. Some charts could not be found or used and were classified as missing data and the corresponding adolescents were not included in the analyses. Sensitivity analyses, coding these records either as disclosed HIV status or not disclosed did not lead to a large variation in our findings.

Second, the HIV disclosure status was reported as a dichotomic variable, while disclosure is a process evolving over time. Thus some adolescents may have been categorized as not informed of their HIV status while they may have been told that they harbour a virus, but not specifying HIV. Some studies, preferably those with a qualitative design, specify that some children are partially disclosed. It refers to the following broad category: “child not fully aware of his/her HIV disease but is suspicious, asks questions to the caregiver about the disease and the drug, and, in many cases, assumes that the drug is a cure” [11]. This status could not have been taken into account in our study with no qualitative data recorded. In addition, the age at disclosure was frequently missing and it was not possible to study this variable as a time-dependant information in the survival analyses.

Third, some of the adolescent lost-to-follow-up might have been unreported deaths. We have addressed this possible misclassification by specifically retrieving and reviewing every patient's chart meeting the definition of loss-to-follow-up in case the charts had been completed after the closure of the database for this analysis.

Finally, due to the retrospective design, we could not address the psychosocial effect of disclosure. A cross-sectional study conducted in New York City demonstrated that Youths (n = 196, mean age 12.7 years) with disclosed HIV status were significantly less anxious than those who had not been told but there were no other differences in psychological functioning [30]. This should be verified in African settings as the caregivers' fear for negative psychosocial outcomes is a common barrier to disclose as discussed previously.

In conclusion, most of HIV-infected adolescents on ART in these West African settings were not aware of their HIV status. However, our study showed a strong beneficial effect of HIV disclosure on retention in care after ART initiation beyond the age of ten. This sample of clinics had initiated different strategies to carry out the HIV disclosure process in the absence of guidelines or specific training. Such initiatives need to be promoted and developed as they may provide individual benefits. For this purpose, they need to be described, standardized, evaluated and shared. Also, further studies should look at the effect on other outcomes such as immunological failure, treatment adherence, virological progression, viral resistance, but also anxiety, depression, school performance, family and social relations and sexual risk behaviours. This would provide deeper understanding of HIV disclosure process in adolescents in resource limited settings in order to tailor age-adequate interventions.
